# Neutrophil Extracellular Traps in Anti-Neutrophil Cytoplasmic Antibody-Associated Vasculitis: Diagnostic and Clinical Significance—A Review of the Current Literature

**DOI:** 10.3390/jcm14113639

**Published:** 2025-05-22

**Authors:** Sylwester Drożdżal, Aleksandra Gomółka, Martyna Opara-Bajerowicz, Marcin Lisak, Urszula Sielicka, Katarzyna Bąk, Jarosław Przybyciński, Wiktoria Feret-Adrabińska

**Affiliations:** 1 Clinic of Nephrology, Transplantology and Internal Medicine, Pomeranian Medical University in Szczecin, 70-204 Szczecin, Poland; 2 Clinic of Internal Medicine, Rheumatology, Diabetology, Geriatrics and Clinical Immunology, Pomeranian Medical University in Szczecin, 70-204 Szczecin, Poland

**Keywords:** vasculitis, NETs, ANCA, ANCA vasculitis, autoantibodies, inflammation

## Abstract

Anti-neutrophil cytoplasmic antibody (ANCA)-associated vasculitis (AAV) is a group of rare but potentially life-threatening autoimmune diseases that affect small to medium-sized blood vessels. Recent evidence highlights the critical role of neutrophil extracellular traps (NETs) in the pathophysiology of AAV. NETs, which are web-like structures composed of DNA and antimicrobial proteins, contribute to vascular damage and immune activation. In patients with AAV, excessive or impaired clearance of NETs can trigger autoantibody production and exacerbate inflammation. This literature review demonstrates the association between NETs and disease activity in AAV. Biomarkers such as MPO-DNA complexes and circulating free DNA can be used to assess disease activity and the response to treatment. Understanding NETosis in the clinical context could improve risk stratification, guide treatment decisions, enable the development of new targeted therapies, and support the development of more precise monitoring tools for AAV treatment.

## 1. AAV—Classification, Symptoms, Pathophysiology

Anti-neutrophil cytoplasmic antibody (ANCA)-associated vasculitis (AAV) represents a group of autoimmune disorders marked by inflammation and damage of small blood to mid-sized vessels, frequently leading to significant tissue injury. The three main clinical phenotypes within this group include granulomatosis with polyangiitis (GPA), microscopic polyangiitis (MPA), and eosinophilic granulomatosis with polyangiitis (EGPA), formerly known as Churg–Strauss syndrome [1]. A hallmark of AAV is the loss of immunological tolerance to intracellular neutrophil components, particularly proteinase 3 (PR3) and myeloperoxidase (MPO) [2]. While capillaries, venules, and arterioles are predominantly affected, inflammation may extend to small arteries and veins as well [3,4] (in Table 1—summary of AAV classification).

A key diagnostic and pathogenic feature is the presence of anti-neutrophil cytoplasmic antibodies (ANCAs) directed against PR3 or MPO. These autoantibodies are strongly associated with the clinical manifestations and organ involvement seen in AAV. Both GPA and MPA can impact a wide range of organ systems, but the respiratory tract and kidneys are most frequently involved [5]. The clinical presentation ranges from life-threatening organ dysfunction to more indolent disease courses [6].

GPA is commonly associated with PR3-ANCA and often presents with upper respiratory tract symptoms (e.g., chronic sinusitis), lower respiratory tract involvement (e.g., pulmonary hemorrhage), granulomatous inflammation, and renal disease. In contrast, MPA typically correlates with MPO-ANCA positivity and lacks granulomatous inflammation, though it often presents with more severe renal impairment and pulmonary capillaritis [7]. EGPA is distinguished by asthma, eosinophilia, and variable vasculitic manifestations. Although some patients with EGPA test positive for MPO-ANCA, the condition shows distinct genetic, immunopathological, and clinical features and is often treated as a separate disease entity [7].

Symptoms associated with necrotizing or granulomatous changes may involve the ears, nose, and throat (ENT) and manifest as chronic rhinitis, sinusitis, or laryngitis. Pulmonary involvement in AAV, particularly in GPA and MPA, may result in pulmonary capillaritis, leading to symptoms such as dyspnea, coughing, and hemoptysis caused by alveolar hemorrhage. Ocular involvement can include orbital granulomas, optic neuritis, or vasculitis of the retinal vessels. Cutaneous manifestations are frequent and manifest as purpuric or petechial rashes, reflecting necrotizing vasculitis of dermal vessels [8].

Renal involvement, a defining feature of AAV, typically manifests as rapidly progressive glomerulonephritis (ANCA-GN). If left untreated, it may progress to acute kidney injury (AKI), chronic kidney disease (CKD), or even end-stage renal disease (ESRD), significantly contributing to patient morbidity and mortality [9]. Pathogenetically, the disease process is initiated by ANCA-mediated activation of neutrophils, which then adhere to endothelial cells via integrins and chemokine signaling [10]. Activated neutrophils release cytotoxic agents, such as reactive oxygen species (ROS) and neutrophil extracellular traps (NETs), which results in endothelial injury. A study by Fuchs et al. demonstrated that the presence of NETs promotes thrombosis even in the absence of any typical thrombophilic factors by serving as a base for the deposition of both platelets and fibrin [11]. Moreover, extravascular damage involves immune cell infiltration, including B cells (producing ANCAs and cytokines), T cells, dendritic cells (presenting antigens), and macrophages (which contribute to fibrosis and chronic inflammation) [12]. From a diagnostic perspective, the detection of PR3-ANCA or MPO-ANCA is typically performed using indirect immunofluorescence (IIF) and enzyme-linked immunosorbent assays (ELISAs). Advances in assay technology, particularly solid-phase immunoassays, have significantly improved both sensitivity and specificity in clinical settings [13].

## 2. Neutrophil Extracellular Traps (NETs)

**Figure 1:**
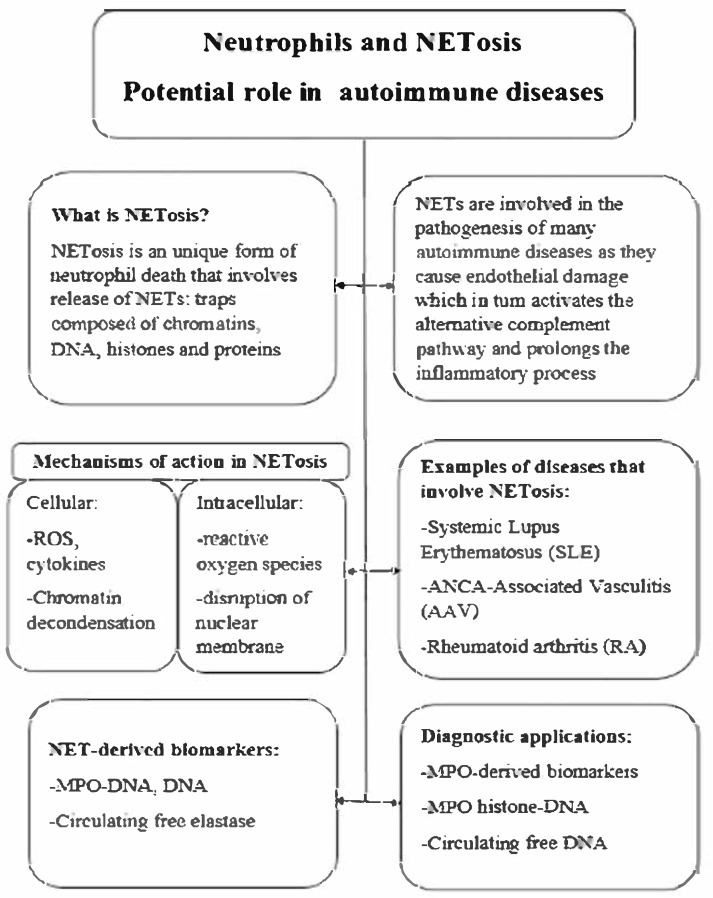
NETosis—potential in autoimmune disease.

**Figure 2:**
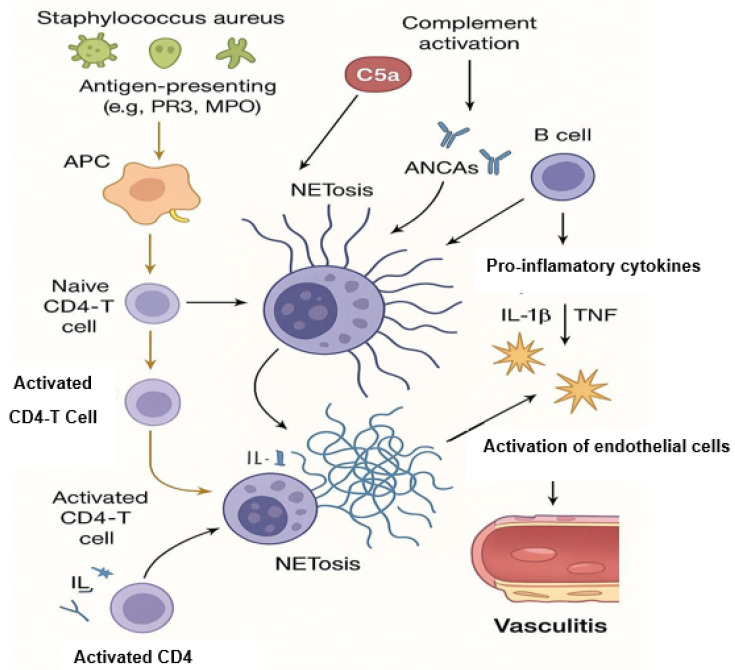
NETosis and its contribution to AAV pathogenesis.

Neutrophil extracellular traps (NETs) represent the antimicrobial mechanism of neutrophils [14]. The basic structure of NETs is extracellular DNA associated with antimicrobial proteins derived from neutrophil granules and the nucleus. The main form of NET formation, called suicide NETosis, leads to neutrophil death and is characterized by subsequent morphological changes: nuclear membrane disintegration, chromatin decondensation, plasma membrane atrophy, and finally leakage of NETs into the extracellular space [15]. In contrast to this mechanism, which leads to neutrophil death, viable NETosis has also been described. In this process, neutrophils remain viable and release only a portion of their nuclear or mitochondrial DNA [16]. NETs have been shown to capture a wide range of microorganisms and provide a key innate immune mechanism. However, excessive NET shedding has been associated with numerous diseases [17]. Although NETs were discovered 21 years ago, the specific signaling events leading to NET release are still largely unclear. Phagocytic NADPH oxidase has been implicated early in this process. Pretreatment of phorbol myristate acetate (PMA)-stimulated neutrophils or Staphylococcus aureus with the NADPH oxidase inhibitor diphenyleneiodonium (DPI) prevented NET release. Addition of an extracellular source of reactive oxygen species (ROS), glucose oxidase, and glucose bypassed the need for NADPH oxidase-generated ROS and induced DPI-independent NET formation in human neutrophils. The best evidence for the requirement of NADPH oxidation for NET extrusion comes from experiments performed in human neutrophils acquired from patients with chronic granulomatous disease (CGD). Patients with CGD have mutations in one of the subunits of the NADPH oxidase enzyme complex, leading to a reduction or absence of the neutrophil respiratory burst [18]. CGD neutrophils do not shed NETs in response to PMA or S. aureus, but release NETs when a NADPH oxidase-independent source of ROS is used. Restoration of NET formation in a patient with X-linked CGD using gp91phox-based gene therapy led to improved clearance of Aspergillus nidulans, underscoring the clinical importance of NADPH oxidase-mediated NETs in antifungal defense [19]. Although NADPH oxidase was originally thought to be essential for NET induction, accumulating evidence has shown that NETs can also be triggered in an NADPH oxidase-independent manner. Both NET release and degradation need to be well-regulated as their accumulation may lead to excessive inflammation. The degradation of NETs is facilitated by DNase1L3, an endonuclease mainly produced in the pancreas. Studies conducted on patients with SLE showed the crucial role of DNase1, as the presence of anti-DNase1 antibodies prevented the degradation of NETs [20]. DNASE1L3 plays a pivotal role in the degradation of extracellular chromatin, particularly within neutrophil extracellular traps (NETs). Deficiency or functional impairment of DNASE1L3 results in the accumulation of immunogenic DNA, thereby contributing to the pathogenesis of systemic autoimmune diseases. Monogenic loss-of-function mutations in DNASE1L3 have been identified in patients with early-onset systemic lupus erythematosus (SLE) and hypocomplementemic urticarial vasculitis syndrome (HUVS), highlighting the gene’s central role in immune tolerance [21]. Furthermore, autoantibodies targeting DNASE1L3 have been detected in sporadic SLE cases and are associated with impaired NET clearance and increased disease activity. These findings suggest that DNASE1L3 deficiency or dysfunction links aberrant NET degradation to the pathology of vasculitis and SLE [22]. Clearance of NETs can be also conducted by monocyte-derived macrophages, in a process that is facilitated by preprocessing of these structures by DNASE1L3 as well as their opsonization by C1q [10]. Any disruption in the regulation processes and accumulation of NETs plays a key role in the pathogenesis of several metabolic diseases [23], autoinflammatory diseases [24], and sepsis [25] and is also closely related to AAV. In vitro and in vivo studies have shown that NETs may be associated with disease-induced thrombosis [26], direct endothelial cell toxicity [27], and vascular injury [28] in AAV patients. The underlying cause for these could be the endothelial damage by NETs produced by ANCA-stimulated neutrophils, which activates the alternative complement pathway, further extending the inflammatory process, and ultimately creating a vicious cycle of neutrophil recruitment and activation (Figure 1). Therefore, NETs may be involved in the occurrence and progression of AAV [29]. Interestingly, when nuclear chromatids in AAV are extruded into the extracellular space of cells as NETs, a new type of cell death is induced—NETosis [30] (Figure 1 and Figure 2). Tao et al. reported that targeting NETosis in various ways can reduce the severity of many diseases and thus improve survival [31,32].

## 3. NETosis and Its Contribution to AAV Pathogenesis

In ANCA-associated vasculitis (AAV), the immunopathogenesis is characterized by a complex interplay between innate and adaptive immune responses, leading to small-vessel inflammation and tissue damage. A hallmark of AAV is the presence of anti-neutrophil cytoplasmic antibodies (ANCAs) targeting neutrophil granule proteins, primarily myeloperoxidase (MPO) and proteinase 3 (PR3). These autoantibodies bind to their respective antigens on the surface of primed neutrophils, leading to their activation. Activated neutrophils undergo a unique form of cell death known as NETosis, releasing neutrophil extracellular traps (NETs) composed of decondensed chromatin and granule proteins, including MPO and PR3. NETs contribute to endothelial damage and perpetuate inflammation by serving as a source of autoantigens, further stimulating ANCA production and creating a self-amplifying cycle of inflammation. The complement system, particularly the alternative pathway, plays a significant role in AAV pathogenesis. NETs can activate the complement cascade, leading to the generation of C5a, a potent chemoattractant that primes neutrophils and enhances their responsiveness to ANCAs. This creates a positive feedback loop, exacerbating vascular injury. Therapeutic interventions targeting the C5a receptor, such as avacopan, have shown promise in modulating this pathway and reducing disease activity. Furthermore, impaired clearance of NETs due to reduced DNase I activity has been observed in AAV patients, leading to the persistence of these pro-inflammatory structures. This accumulation of NETs not only sustains inflammation but also promotes the presentation of autoantigens to the adaptive immune system, facilitating the production of ANCAs. The involvement of receptor-interacting protein kinases (RIPK3) and cyclophilin D (CypD) in NET formation suggests that necroptosis, a form of programmed necrosis, may also contribute to disease pathogenesis. Collectively, these findings underscore the central role of NETosis and complement activation in the immunopathogenesis of AAV, highlighting potential therapeutic targets aimed at disrupting this deleterious cycle [33,34,35].

Studies have demonstrated that ANCAs can induce NETosis, and the propensity for NET formation correlates with disease activity in AAV patients. NETs contribute to vascular damage by promoting endothelial injury and inflammation. Moreover, impaired clearance of NETs due to reduced DNase I activity has been observed in AAV, leading to the persistence of these pro-inflammatory structures [36].

Wang et al. (2016) [37] measured serum DNase I activity in patients with AAV and found significantly reduced levels compared to healthy controls (0.22 ± 0.11 U/mL vs. 0.31 ± 0.12 U/mL, *p* = 0.021), suggesting impaired degradation of extracellular DNA. Furthermore, a negative correlation between DNase I activity and cell-free DNA levels (r = −0.499, *p* = 0.021) supported the hypothesis that reduced DNase activity contributes to NET accumulation and inflammation in AAV [37].

The activation of the complement system, particularly the alternative pathway, plays a significant role in AAV. Xiao et al. (2007) demonstrated that the alternative complement pathway is essential for the development of AAV in animal models [38]. Furthermore, Konwar et al. (2025) identified thrombospondin-1 as an inhibitor of the alternative complement pathway, suggesting potential therapeutic avenues for modulating complement activation in AAV [39].

## 4. Detection of NETosis—Methods Overview

Neutrophil extracellular traps (NETs) play a crucial role in the immune response, but their dysregulation is implicated in various pathologies. Detecting NETosis requires diverse methodologies tailored to the biological context and research goals [24,25].

The most common techniques include immunofluorescence microscopy, which enables morphological visualization of NETs using specific antibodies (e.g., against citrullinated histone H3 or myeloperoxidase), and ELISA-based assays, such as MPO-DNA or CitH3-DNA complexes, which provide quantitative plasma or serum measurements. While immunofluorescence offers high specificity, ELISA allows high-throughput screening, albeit with limitations in distinguishing NETs from other forms of cell-free DNA [40,41].

Fluorescence-based DNA dyes like Sytox Green are also employed to track extracellular DNA in real time, although their specificity for NETs is limited. Flow cytometry allows for multiparametric analysis of neutrophil surface or intracellular markers, but may miss structural NETs. Confocal microscopy offers a superior resolution and 3D visualization, useful for co-localization studies. Finally, machine learning algorithms are increasingly used to automate NET detection in microscopy datasets, enhancing reproducibility and throughput [42,43].

Each method varies in terms of sensitivity, specificity, and applicability, and combining techniques is often essential for robust NETosis analysis (summary in Table 2) [44].

## 5. Therapeutic Implications and Future Directions

**Figure 3:**
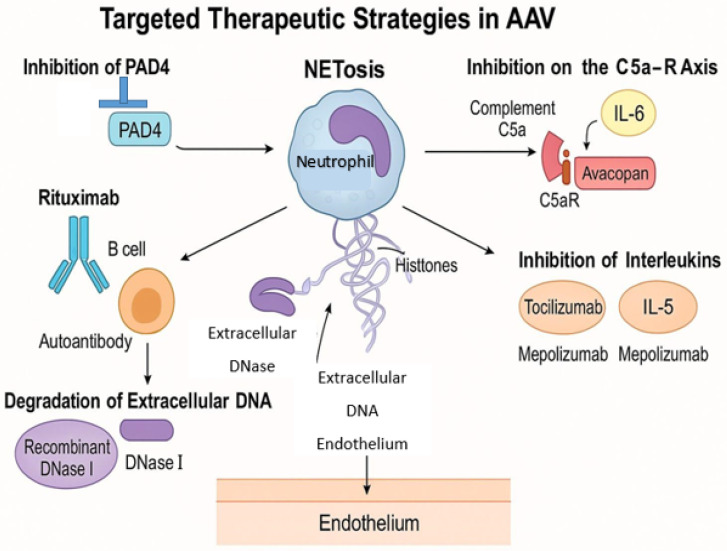
Target therapeutic strategies in AAV.

The recognition of neutrophil extracellular traps (NETs) as pivotal contributors to the pathogenesis of ANCA-associated vasculitis (AAV) has opened up new paths for targeted therapeutic strategies (Figure 3). Traditional treatments, including high-dose glucocorticoids and broad-spectrum immunosuppressants like cyclophosphamide, have been effective but are associated with significant adverse effects and high relapse rates. Consequently, there is a pressing need for therapies that specifically target pathogenic mechanisms such as NETosis [33,45].

The complement system, particularly the C5a-C5a receptor (C5aR) axis, plays a crucial role in neutrophil activation and NET formation. Avacopan, an oral C5aR antagonist, has demonstrated efficacy in reducing disease activity and steroid dependence in AAV patients. Clinical trials have shown that avacopan, in combination with standard therapy, achieved sustained remission with fewer glucocorticoid-related side effects. Avacopan, an oral C5a receptor antagonist, has been approved for the treatment of AAV. In the ADVOCATE trial, avacopan demonstrated non-inferiority to prednisone in inducing remission at 26 weeks and superiority in sustaining remission at 52 weeks (66% vs. 55%; *p* = 0.007). Additionally, avacopan-treated patients experienced fewer glucocorticoid-related adverse events. By inhibiting the C5a-C5aR axis, avacopan reduces neutrophil activation and subsequent NET formation [46].

B cells are instrumental in ANCA production, and their depletion has been a focus of therapeutic intervention. Rituximab, an anti-CD20 monoclonal antibody, has been established as an effective treatment for inducing and maintaining remission in AAV. By reducing ANCA levels, rituximab indirectly diminishes NET formation. However, some patients may experience relapse, which indicates the need for additional or alternative therapies [47,48].

Targeting the NETosis pathway directly offers a promising therapeutic strategy. Agents that inhibit peptidylarginine deiminase 4 (PAD4), an enzyme critical for chromatin decondensation during NET formation, are undergoing investigation. Additionally, recombinant DNase I therapy aims to degrade extracellular DNA in NETs, potentially reducing their pro-inflammatory effects. While these approaches have shown efficacy in preclinical models, clinical trials are necessary to establish their safety and effectiveness in humans [49,50].

Cytokines and chemokines are integral to the inflammatory milieu in AAV. Therapies targeting interleukins such as IL-6 (e.g., tocilizumab) and IL-5 (e.g., mepolizumab) have been explored, particularly in eosinophilic granulomatosis with polyangiitis (EGPA), a subset of AAV. These agents may modulate immune responses and reduce NET formation, although their roles in other AAV subtypes require further elucidation [51,52]. Mepolizumab, targeting interleukin-5 (IL-5), has been evaluated in eosinophilic granulomatosis with polyangiitis (EGPA), a subset of AAV. In the MIRRA trial, 53% of patients receiving mepolizumab achieved remission compared to 19% in the placebo group (*p* < 0.001). While the study did not directly assess NET levels, IL-5 is known to influence eosinophil activity and may indirectly affect NET formation [53].

Advancements in understanding the molecular underpinnings of AAV have paved the way for personalized medicine approaches. Identifying biomarkers associated with NETosis, such as circulating cell-free DNA or specific histone modifications, could aid in disease monitoring and treatment stratification. Tailoring therapies based on individual patient profiles may enhance treatment efficacy and minimize adverse effects [13,54].

Another emerging strategy in NET-targeted therapy involves monoclonal antibodies that neutralize extracellular histones, key components of NETs responsible for cytotoxicity and inflammation. CIT-013 (developed by Cyclica, formerly known as ANp3A by Cytrill) is a first-in-class monoclonal antibody designed to selectively bind citrullinated histones H2A and H4, which are released during NETosis. By targeting these modified histones, CIT-013 can inhibit the formation of NETs and promote the clearance of existing NET structures through Fc-mediated phagocytosis. Preclinical studies have shown that CIT-013 not only reduces NET burden but also alleviates tissue damage and inflammation in models of rheumatoid arthritis and other autoimmune conditions. Immunohistochemistry of synovial biopsies from RA patients has confirmed the presence of CIT-013 target epitopes in areas of active inflammation, supporting the clinical relevance of this therapeutic approach [41,55].

As a result, therapeutic strategies targeting NETosis are being actively explored for their potential to modulate disease activity and improve clinical outcomes in AAV patients.

The following table (Table 3) summarizes selected clinical trials investigating therapies that affect NETosis, either directly or indirectly, in the context of AAV. These include agents such as CIT-013, a monoclonal antibody targeting citrullinated histones, and avacopan, a C5a receptor antagonist that modulates neutrophil activation and may influence NET formation [46].

## 6. Summary

Anti-neutrophil cytoplasmic antibody (ANCA)-associated vasculitis (AAV) is a group of autoimmune diseases characterized by inflammation of small to medium-sized blood vessels. Neutrophil extracellular traps (NETs)—networks of DNA and antimicrobial proteins expelled by neutrophils—play a crucial role in the pathogenesis of AAV.

The recent literature indicates that NETs contribute to vascular damage and autoantigen exposure, promoting the generation of ANCAs. In AAV patients, impaired degradation of NETs and increased NET formation have been observed, which sustain the inflammatory process and lead to endothelial injury.

NET-related biomarkers, such as MPO-DNA complexes, citrullinated histones, and cell-free DNA, are being investigated as potential diagnostic and prognostic indicators in AAV. These markers may help assess disease activity, predict flares, and monitor treatment response.

Understanding the role of NETs in AAV has therapeutic implications: Targeting NET formation (NETosis) or promoting their degradation could be a novel therapeutic strategy. Agents like PAD4 inhibitors, DNase, or ROS inhibitors are being explored in preclinical and early clinical studies. Traditional immunosuppressants (e.g., cyclophosphamide, rituximab) also indirectly reduce NET formation by suppressing neutrophil activation.

## 7. Conclusions

NETs are central to the immunopathology of ANCA-associated vasculitis. Their dual role as both mediators of tissue damage and biomarkers of disease activity makes them a promising focus for future diagnostics and therapies. Further research is necessary to translate these findings into routine clinical practice.

## Figures and Tables

**Table 1:** Classification of ANCA-associated vasculitis (AAV) [3].

Subtype	ANCA Specificity	Key Clinical Features	Histopathology	Commonly Affected Organs
Granulomatosis with Polyangiitis (GPA)	PR3-ANCA (c-ANCA); occasionally MPO-ANCA	- Necrotizing granulomatous inflammation - Upper and lower respiratory tract involvement - Necrotizing glomerulonephritis - Nasal crusting, sinusitis, pulmonary nodules	- Necrotizing granulomatous inflammation - Pauci-immune necrotizing vasculitis	Upper/lower respiratory tract, kidneys, eyes, skin, peripheral nerves
Microscopic Polyangiitis (MPA)	MPO-ANCA (p-ANCA); occasionally PR3-ANCA	- Rapidly progressive glomerulonephritis - Pulmonary capillaritis - Purpura, mononeuritis multiplex - No granulomatous inflammation	- Pauci-immune necrotizing vasculitis - Necrotizing glomerulonephritis - No granulomas	Kidneys, lungs, skin, peripheral nerves
Eosinophilic Granulomatosis with Polyangiitis (EGPA)	MPO-ANCA (p-ANCA) in ~30–40%; often ANCA-negative	- Asthma, allergic rhinitis - Eosinophilia - Pulmonary infiltrates - Cardiac and nerve involvement	- Eosinophil-rich necrotizing granulomas - Necrotizing vasculitis	Lungs, nerves, heart, skin, GI tract
ANCA-Negative Vasculitis	None detected	- Similar to other AAV subtypes - Diagnosis based on clinical and histopathological findings	- Findings similar to AAV - ANCA absent	Varies depending on clinical features

**Table 2:** Methods for detecting NETosis.

Method	Principle	Advantages	Limitations
Immunofluorescence Microscopy	Visualization of NETs using fluorescent antibodies targeting NET components (e.g., CitH3, MPO)	High specificity; allows morphological assessment; can be semi-quantitative	Time-consuming; requires specialized equipment and expertise
ELISA (e.g., MPO-DNA, CitH3-DNA)	Quantification of NET components in plasma/serum using antibody-based detection	Quantitative; suitable for high-throughput analysis; relatively simple to perform	Potential cross-reactivity; may not distinguish NETs from other sources of cell-free DNA
Sytox Green Assay	Detection of extracellular DNA by fluorescence upon binding to DNA	Rapid; cost-effective; suitable for live-cell imaging	Not specific to NETs; cannot differentiate between NETosis and other forms of cell death
Flow Cytometry	Measurement of NET-associated markers on neutrophils using fluorescent antibodies	Quantitative; allows analysis of large cell populations; can assess multiple markers simultaneously	Requires cell suspension; may not detect NET structures effectively
Confocal Microscopy	High-resolution imaging of NETs in three dimensions	Detailed structural analysis; can confirm co-localization of NET components	Expensive equipment; lower throughput compared to other methods
Machine Learning-Based Analysis	Automated identification and quantification of NETs using trained algorithms on imaging data	High-throughput; reduces observer bias; can handle large datasets	Requires computational resources and expertise; dependent on quality of training data

**Table 3:** Selected clinical trials targeting NETosis s in ANCA-associated vasculitis (AAV).

Phase	Study Design	Population	Key Findings	Source
Phase I	Randomized, double-blind, placebo-controlled	Healthy volunteers	CIT-013 was well-tolerated up to 0.3 mg/kg IV and 0.9 mg/kg with premedication. Subcutaneous administration showed good bioavailability (~66%) and was well-tolerated. Near-complete inhibition of LPS-induced NETs observed at 0.3 and 0.9 mg/kg doses.	https://acrabstracts.org/abstract/early-clinical-development-of-cit-013-a-first-in-class-netosis-inhibitor-in-a-randomized-phase-i-dose-escalation-study-in-healthy-volunteers-demonstrating-potent-inhibition-of-lps-induced-neutrophil/ (accessed on 27 April 2025)
Phase IIa (Planned)	Proof-of-concept	RA and HS patients	Phase IIa studies are planned to commence in 2025 to evaluate efficacy in RA and HS.	https://citryll.com/cit-013-clinical-development/ (accessed on 27 April 2025)

## Data Availability

No new data were created or analyzed in this study.
